# Supplementary material for: MGP-AttTCN: An interpretable machine learning model for the prediction of sepsis
Source: PLoS One. 2021 May 7;16(5):e0251248. doi: 10.1371/journal.pone.0251248 (PMC8104377; doi:10.1371/journal.pone.0251248)
Supplement: S1 File — (PDF) [file pone.0251248.s001.pdf]

S1 Table. Area under the ROC curve for our labels.

Time to onset	MGP-Log.Reg.	MGP-TCN	MGP-AttTCN w/o α	MGP-AttTCN w/o β	MGP-AttTCN w/ SE kernel	MGP-AttTCN
6h	65.2 ± 1.2	63.4 ± 1.6	**66.7 ± 1.1**	56.6 ± 1.1	66.1 ± 1.4	65.7 ± 1.6
5h	64.3 ± 1.3	63.5 ± 1.2	66.3 ± 1.4	54.1 ± 1.0	65.9 ± 1.2	**66.0 ± 1.1**
4h	66.1 ± 0.9	63.2 ± 1.3	66.2 ± 1.3	56.6 ± 1.4	64.2 ± 1.1	**66.4 ± 1.3**
3h	65.1 ± 0.8	64.4 ± 1.0	65.4 ± 0.8	57.0 ± 1.2	63.4 ± 1.0	**65.7 ± 1.2**
2h	**67.0 ± 0.7**	65.5 ± 1.0	66.4 ± 1.0	57.6 ± 0.9	63.4 ± 0.8	66.5 ± 1.1
1h	**68.5 ± 0.6**	66.7 ± 1.3	67.9 ± 1.0	59.1 ± 0.8	64.9 ± 0.8	66.6 ± 0.8
0h	67.5 ± 0.4	67.3 ± 0.9	**67.7 ± 0.7**	62.1 ± 0.8	65.5 ± 0.6	65.9 ± 0.6

S2 Table. Area under the ROC curve for Moor et al. [21] labels.

Time to onset	MGP-Log.Reg.	MGP-TCN	MGP-AttTCN w/o α	MGP-AttTCN w/o β	MGP-AttTCN w/ SE kernel	MGP-AttTCN
6h	72.7 ± 1.4	73.5 ± 1.2	67.3 ± 1.9	70.1 ± 3.1	70.7 ± 1.4	**76.7 ± 1.4**
5h	73.6 ± 1.3	74.9 ± 0.7	67.6 ± 1.0	69.3 ± 2.2	70.1 ± 1.7	**76.3 ± 1.0**
4h	75.2 ± 1.3	**74.9 ± 1.0**	68.0 ± 0.9	72.3 ± 2.5	70.5 ± 0.9	74.6 ± 1.5
3h	76.8 ± 1.2	76.0 ± 0.6	69.9 ± 1.4	72.6 ± 1.6	72.1 ± 1.3	76.3 ± 0.9
2h	79.4 ± 0.6	**80.2 ± 0.6**	73.6 ± 1.1	76.3 ± 2.0	75.2 ± 0.8	78.9 ± 1.4
1h	82.6 ± 0.5	**83.6 ± 0.4**	77.3 ± 0.9	78.1 ± 1.6	80.5 ± 0.6	82.0 ± 0.8
0h	83.5 ± 0.4	**87.0 ± 0.5**	76.9 ± 0.7	79.3 ± 1.0	83.6 ± 0.6	82.3 ± 0.7

S3 Table. Area under the precision-recall curve for our labels.

Time to onset	MGP-Log.Reg.	MGP-TCN	MGP-AttTCN w/o α	MGP-AttTCN w/o β	MGP-AttTCN w/ SE kernel	MGP-AttTCN
6h	47.0 ± 1.5	47.3 ± 1.9	**50.5 ± 1.6**	41.4 ± 1.1	49.1 ± 1.7	48.4 ± 1.4
5h	45.3 ± 1.3	46.0 ± 1.1	**49.6 ± 1.4**	38.1 ± 1.3	47.5 ± 1.1	48.3 ± 1.9
4h	48.5 ± 1.1	46.5 ± 1.6	**49.4 ± 2.0**	42.2 ± 1.5	48.3 ± 1.2	48.8 ± 1.4
3h	**49.1 ± 1.0**	47.5 ± 1.0	49.1 ± 1.1	42.4 ± 1.2	48.1 ± 1.1	48.3 ± 1.2
2h	**50.8 ± 0.8**	48.9 ± 1.3	49.2 ± 1.2	43.2 ± 0.8	50.2 ± 1.1	48.6 ± 0.9
1h	**52.3 ± 0.8**	50.1 ± 1.5	51.7 ± 1.2	44.7 ± 0.9	51.2 ± 0.8	49.0 ± 1.0
0h	55.8 ± 0.6	53.9 ± 1.2	55.7 ± 0.8	48.6 ± 1.1	**57.1 ± 0.8**	52.2 ± 0.7

S4 Table. Area under the precision-recall curve for Moor et al. [21] labels.

Time to onset	MGP-Log.Reg.	MGP-TCN	MGP-AttTCN w/o α	MGP-AttTCN w/o β	MGP-AttTCN w/ SE kernel	MGP-AttTCN
6h	**29.2 ± 2.0**	20.9 ± 1.1	23.4 ± 1.6	22.8 ± 2.5	24.2 ± 2.4	24.2 ± 1.4
5h	**28.3 ± 1.9**	22.3 ± 1.2	23.9 ± 1.7	21.4 ± 2.2	27.0 ± 3.0	24.2 ± 1.4
4h	**29.3 ± 2.0**	22.9 ± 1.3	23.1 ± 1.4	22.6 ± 1.6	26.6 ± 1.9	25.5 ± 1.4
3h	**29.7 ± 1.2**	24.5 ± 1.0	24.5 ± 1.3	23.6 ± 1.5	26.5 ± 1.5	28.6 ± 1.7
2h	**36.7 ± 1.8**	31.4 ± 1.6	29.3 ± 1.2	28.1 ± 1.8	33.2 ± 1.4	31.5 ± 2.3
1h	**40.5 ± 0.9**	36.8 ± 1.0	32.5 ± 1.4	31.6 ± 1.9	38.2 ± 1.4	39.4 ± 1.6
0h	44.4 ± 1.3	**47.0 ± 1.3**	33.8 ± 1.0	36.8 ± 1.1	41.0 ± 1.1	43.1 ± 1.6

S5 Table. Area under the ROC curve for our labels and the baseline dataset.

Time to onset	Log. Reg.	InSight	MGP-AttTCN
6h	57.2	54.7	**64.05 ± 2.2**
5h	56.6	49.0	**66.36 ± 1.95**
4h	53.8	55.9	**67.46 ± 1.59**
3h	53.2	53.1	**66.52 ± 1.65**
2h	53.8	55.2	**66.99 ± 1.62**
1h	54.3	57.3	**66.39 ± 1.28**
0h	50.8	55.9	**64.66 ± 1.82**

S6 Table. Area under the ROC curve for Moor et al. [21] labels and the baseline dataset.

Time to onset	Log. Reg.	InSight	MGP-AttTCN
6h	**73.9**	64.5	72.58 ± 1.82
5h	**73.9**	64.4	72.5 ± 1.59
4h	**76.2**	65.7	71.78 ± 1.42
3h	**76.5**	74.1	73.36 ± 1.19
2h	**76.2**	69.7	75.39 ± 1.55
1h	77.0	74.1	**77.71 ± 1.33**
0h	77.3	73.1	**78.56 ± 1.28**

S7 Table. Area under the Precision-Recall curve for our labels and the baseline dataset.

Time to onset	Log. Reg.	InSight	MGP-AttTCN
6h	37.7	41.4	**41.68 ± 2.27**
5h	37.6	35.9	**43.29 ± 2.5**
4h	37.9	45.2	**47.7 ± 2.22**
3h	36.2	38.2	**45.81 ± 2.44**
2h	39.9	38.8	**46.09 ± 1.71**
1h	42.2	41.8	**47.81 ± 1.75**
0h	40.7	46.3	**49.31 ± 1.78**

S8 Table. Area under the Precision-Recall curve for Moor et al. [21] labels and the baseline dataset.

Time to onset	Log. Reg.	InSight	MGP-AttTCN
6h	**44.5**	37.2	35.52 ± 2.05
5h	**44.3**	37.2	38.43 ± 2.51
4h	**50.9**	40.9	38.54 ± 1.51
3h	**55.2**	52.2	42.07 ± 1.22
2h	**53.7**	48.4	47.08 ± 1.86
1h	**55.8**	52.2	53.09 ± 1.19
0h	55.7	53.9	**57.87 ± 1.86**

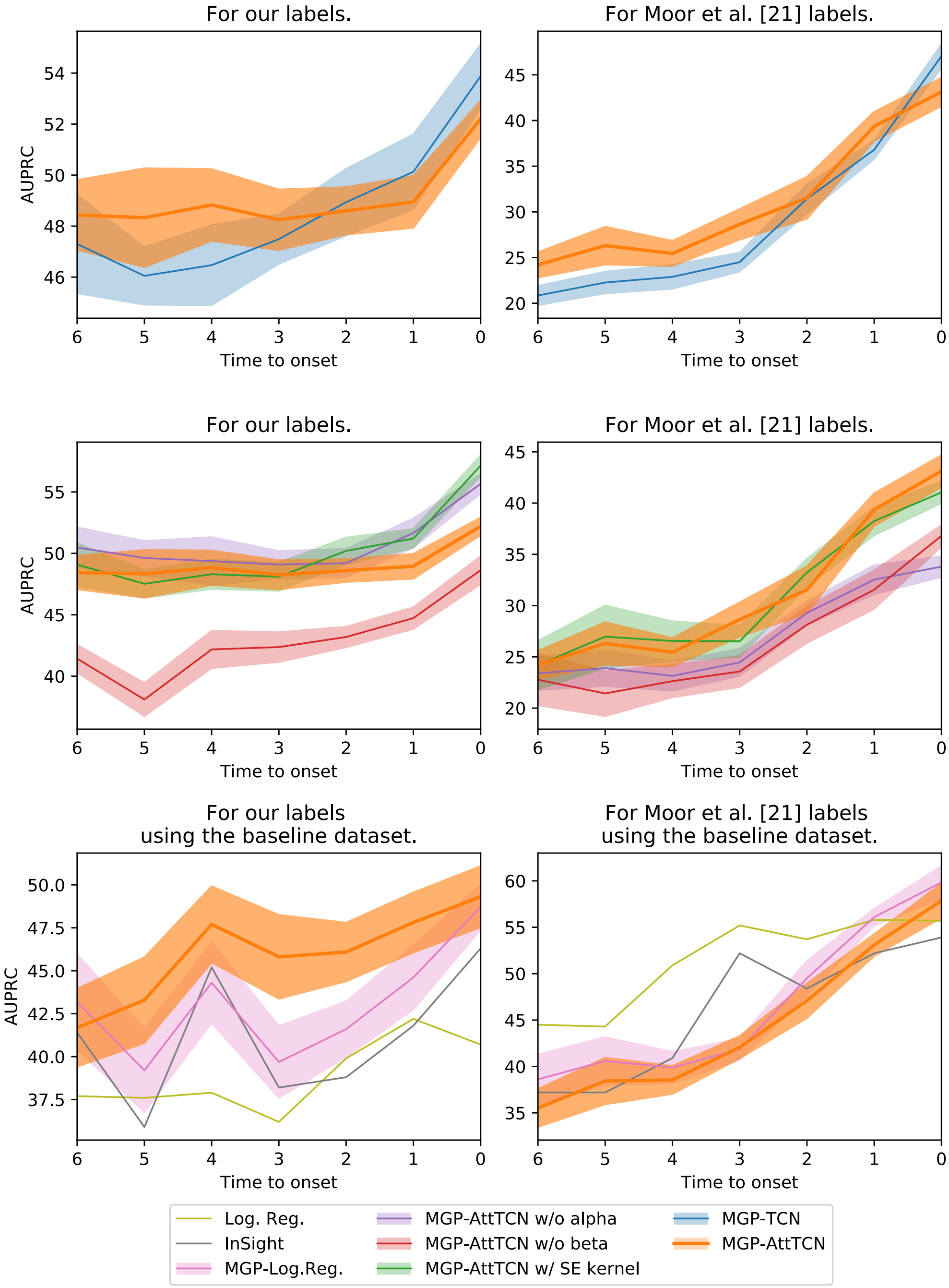

S1 Fig. Area under the Precision-Recall curve of different models.
